# Microglia lacking a peroxisomal β-oxidation enzyme chronically alter their inflammatory profile without evoking neuronal and behavioral deficits

**DOI:** 10.1186/s12974-019-1442-3

**Published:** 2019-03-13

**Authors:** Lien Beckers, Ivana Geric, Stijn Stroobants, Sander Beel, Philip Van Damme, Rudi D’Hooge, Myriam Baes

**Affiliations:** 1 Department of Pharmaceutical and Pharmacological Sciences, Laboratory for Cell Metabolism, KU Leuven - University of Leuven, Campus Gasthuisberg O/N2, Herestraat 49, B-3000 Leuven, Belgium; 2 Faculty of Psychology and Educational Sciences, Biological Psychology Unit, KU Leuven - University of Leuven, B-3000 Leuven, Belgium; 3 Department of Neurosciences, Laboratory for Neurobiology, KU Leuven - University of Leuven, Leuven, Belgium; 4 Center for Brain and Disease Research, VIB, Leuven, Belgium; 5 Neurology Department, University Hospitals Leuven, Leuven, Belgium; 6 Present Address: Center for Translational and Computational Neuro-immunology, Department of Neurology, Columbia University Medical Center, New York City, NY USA

**Keywords:** Microglia, Peroxisomes, Facial nerve axotomy, Conditional mouse model, β-Oxidation, Behavior, Immune response

## Abstract

**Background:**

Microglia play a central role in most neurological disorders, but the impact of microgliosis on brain environment and clinical functions is not fully understood. Mice lacking multifunctional protein-2 (MFP2), a pivotal enzyme in peroxisomal β-oxidation, develop a fatal disorder characterized by motor problems similar to the milder form of MFP2 deficiency in humans. The hallmark of disease in mice is the chronic proliferation of microglia in the brain, but molecular pathomechanisms that drive rapid clinical deterioration in human and mice remain unknown. In the present study, we identified the effects of specific deletion of MFP2 from microglia in the brain on immune responses, neuronal functioning, and behavior.

**Methods:**

We created a novel *Cx3cr1-Mfp2*^*−/−*^ mouse model and studied the impact of MFP2 deficiency on microglial behavior at different ages using immunohistochemistry and real-time PCR. Pro- and anti-inflammatory responses of *Mfp2*^*−/−*^ microglia were assessed in vitro and in vivo after stimulation with IL-1β/INFγ and IL-4 (in vitro) and LPS and IL-4 (in vivo). Facial nerve axotomy was unilaterally performed in *Cx3cr1-Mfp2*^*−/−*^ and control mice, and microglial functioning in response to neuronal injury was subsequently analyzed by histology and real-time PCR. Finally, neuronal function, motor function, behavior, and cognition were assessed using brainstem auditory evoked potentials, grip strength and inverted grid test, open field exploration, and passive avoidance learning, respectively.

**Results:**

We found that *Mfp2*^*−/−*^ microglia in a genetically intact brain environment adopt an inflammatory activated and proliferative state. In addition, we found that acute inflammatory and neuronal injury provoked normal responses of *Mfp2*^*−/−*^ microglia in *Cx3cr1-Mfp2*^*−/−*^ mice during the post-injury period. Despite chronic pro-inflammatory microglial reactivity, *Cx3cr1-Mfp2*^*−/−*^ mice exhibited normal neuronal transmission, clinical performance, and cognition.

**Conclusion:**

Our data demonstrate that MFP2 deficiency in microglia causes intrinsic dysregulation of their inflammatory profile, which is not harmful to neuronal function, motor function, and cognition in mice during their first year of life.

**Electronic supplementary material:**

The online version of this article (10.1186/s12974-019-1442-3) contains supplementary material, which is available to authorized users.

## Background

Inactivation of peroxisomal β-oxidation by the loss of multifunctional protein-2 (MFP2) in human and mice causes a fatal neuropathological phenotype [1–4]. MFP2, encoded by the *Hsd17b4* gene, is the key enzyme in peroxisomal β-oxidation, a pathway responsible for chain shortening of carboxylates including very long chain fatty acids and formation of polyunsaturated fatty acids [5]. Dependent on the type of mutation, patients with MFP2 (also called D-bifunctional protein) deficiency display a severe neurodevelopmental disorder leading to death within the first year of life or a milder phenotype with prolonged survival into adolescence or adulthood [3, 6, 7]. Prominent clinical presentations of the milder phenotype are sensorineural hearing loss, leukodystrophy, intellectual decline, ataxia, and sensorimotor neuropathy [3, 8, 9]. Most symptoms are mimicked by the constitutive *Mfp2*^*−/−*^ mouse model which develops a progressive fatal phenotype characterized by motor problems, ataxia, weight loss, and lethargy [1, 2]. The pathomechanisms of disease and role of MFP2 in the brain remain however elusive in human and mice.

The most prominent hallmark of *Mfp2*^*−/−*^ mice is a strong neuroinflammatory response consisting of proliferating resident microglia in the absence of neuronal loss [2, 10, 11]. Characterization of this excessive microgliosis in the brain of *Mfp2*^*−/−*^ mice revealed that resident microglia proliferate, adopt a permanently activated non-phagocytic state, and lose their homeostatic signature [10, 11]. Specific suppression of microgliosis in *Mfp2*^*−/−*^ mice by treatment with PLX5622, a selective colony-stimulating factor 1 receptor (CSF1R) inhibitor, failed to prevent neuronal dysfunction and clinical deterioration of *Mfp2*^*−/−*^ mice as inflammatory responses and residual reactive microglia remained after treatment [12]. The importance of peroxisomal β-oxidation in innate immune cells is poorly understood, but *Mfp2*^*−/−*^ mice do not show systemic inflammation, and there is no infiltration of peripheral immune cells in the brain [10].

Microglia, the primary immune effector cells in the brain, can rapidly respond to disturbances of central nervous system (CNS) homeostasis by adopting an inflammatory activation state which consists of morphological alterations, proliferation, upregulation of cell surface markers, and increased expression of inflammatory molecules [13–15]. The so-called guardians of the brain adopt resting and activated states depending on the brain environment or the insult. Chronic activation of microglia was assumed to be detrimental to proper CNS functioning, but microglial activation is in fact a delicately balanced process that constitutes both harmful and protective effects [16–18].

This early-onset aberrant phenotype of *Mfp2*^*−/−*^ microglia gains even more interest as we previously defined that microglia in *Nestin-Mfp2*^*−/−*^ mice develop a late-onset and mild inflammatory state [11]. The neural-specific *Nestin-Mfp2*^*−/−*^ mouse model lacks MFP2 in neurons, astrocytes, and oligodendrocytes but not in microglia [2]. The chronic and strongly activated microglial phenotype in constitutive *Mfp2*^*−/−*^ mice was associated with early-onset deficits in neuronal transmission, explorative behavior, and cognition. In contrast, attenuated microgliosis in *Nestin*-*Mfp2*^*−/−*^ mice was associated with late-onset and minor abnormalities in neuronal function and behavior compared to *Mfp2*^*−/−*^ mice. Whereas constitutive *Mfp2*^*−/−*^ mice die within 4–6 months, *Nestin-Mfp2*^*−/−*^ mice survive up to 8–12 months [2, 19]. Although the progression of microgliosis parallels clinical deterioration, it remains unknown whether the dysregulated microglial phenotype and the behavioral abnormalities are caused by cell-autonomous MFP2 dysfunction in microglia.

Therefore, to investigate the importance of MFP2 function within microglia, we generated a novel mouse model that lacks MFP2 specifically in myeloid cells by *Cx3cr1-*driven recombination of the *Hsd17b4* gene [20]. In the brain parenchyma, the chemokine receptor CX3CR1 is exclusively expressed by microglia [21, 22]. We characterized the *Cx3cr1*-*Mfp2*^*−/−*^ mice with regard to the morphological and immunological properties of microglia and examined responses of *Mfp2*^*−/−*^ microglia in vitro and in vivo to immunological challenges and neuronal injury by facial nerve axotomy. In addition, the impact of microglia-specific deletion of MFP2 on neuronal functioning and murine behavior and cognition was assessed. Our study demonstrated that microglia-specific deletion of MFP2 from the *Cx3cr1-Mfp2*^*−/−*^ brain leads to intrinsic alterations of microglia that develop a pro-inflammatory and proliferative phenotype but retain proper responses to inflammatory stimuli. This chronic adaptation of *Mfp2*^*−/−*^ microglia in a genetically intact brain did however not affect neuronal transmission or murine motor function, cognition, and explorative behavior within the time frame wherein all *Mfp2*^*−/−*^ mice have died from the disease [19].

## Methods

### Mouse breeding

*Mfp2*^*loxP/loxP*^ mice in which exon 8 of the *Hsd17b4* gene is flanked by LoxP sequences [2] were bred with *Cx3cr1-Cre* mice, which cause recombination in brain microglia, monocytes, subsets of natural killer, and dendritic cells [20], on a C57Bl6 background. *Mfp2*^*−/−*^ mice were obtained by breeding heterozygous mice as described [2]. Genotyping was performed on ear punches. All mice were bred in the animal housing facility of the KU Leuven, had ad libitum access to water and standard rodent food, and were kept on a 12-h light and dark cycle.

### Murine behavioral studies

The auditory brainstem response test (BAEP), the open field (OF) exploration, and the grip strength measurement were conducted as previously described [11]. Passive avoidance (PA) learning was examined in a cage consisting of a light and a dark compartment containing a grid floor [23]. After a 30-min adaptation to the dark, the mouse was placed in the light compartment for a training trial. After 5 s, the dark compartment was opened and step-through latency was manually recorded. When all paws were placed on the grid floor, a mild electric footshock (0.2 mA, 2 s) was applied. Retention was tested 24 h later in the dark-adapted mouse, and latency to enter the dark compartment was measured up to a 300-s cutoff value. The inverted grid test or four-limb hang test is a test of combined forepaw and hind paw strength and coordination. Mice are placed on a wired grid which is subsequently inverted. The latency to fall is recorded with a time limit of 300 s. In general, normal mice are able to remain on the inverted grid for at least 300 s. Mice that fall off the grid before the time limit of 300 s were directly given another try, and the best time was recorded. Mice that hang for the 300-s limit were placed back into the cage.

### Administration of lipopolysaccharide

Mice aged 5 months and 8 months received an intraperitoneal (i.p.) injection of lipopolysaccharide (LPS) at a dose of 1 mg/kg (Sigma, L4391) or sterile saline vehicle in a total volume of 100 μl. Four hours later, mice were sacrificed, and brainstem was collected and flash frozen in liquid nitrogen for subsequent RNA analysis.

### Intracerebroventricular injection of IL-4

The IL-4 injections were performed on 5-month-old mice as described previously [24]. Briefly, mice were anesthetized with ketamine and xylazine (100 and 10 mg/kg, respectively) and injected with vehicle (0.9% NaCl) or 200 ng IL-4 (R&D) in a total volume of 2.5 μl in the third cerebral ventricle using the following stereotaxic coordinates: bregma, − 0.25 mm; lateral, 1 mm; and depth, 2.25 mm. The animals were allowed to recover for 20 h, after which they were sacrificed with an overdose of Domitor and Nimatek (1 mg and 75 mg/kg, respectively). The frontal cortex contralateral to the injection site was collected and snap frozen in liquid nitrogen.

### Facial nerve axotomy

The facial nerve injury experiment was performed as described [25]. Mice were 3-month-old at the time of the surgical procedure. In brief, mice were anesthetized with 3% isoflurane and placed on a 37 °C hot plate during the surgical procedure. Unilateral facial nerve transection at the stylomastoid foramen, posterior from the retroauricular branch point, was performed in *Cx3cr1-Mfp2*^*−/*−^ and control mice. The successful outcome of the procedure was verified by ipsilateral whisker paresis immediately upon recovery from mild anesthesia. The facial nerve motor nucleus at the ipsi- and contralateral side of the injury was collected from frozen sections as described [25]. For RNA extraction, the PicoPure RNA isolation kit (Thermo Fisher Scientific) was used.

### Immunohistochemical staining and quantification

Mice were anesthetized with a mix of Domitor (1 mg/kg) and Nimatek (75 mg/kg). Tissue processing and IHC staining were performed as described [1, 26, 27]. Briefly, mice were perfused transcardially with PBS (pH 7.4) followed by 4% paraformaldehyde (PFA). The brains were isolated, post-fixed with 4% PFA overnight, and kept in 70% ethanol prior to paraffin embedding. Paraffin sections (7 μm) were used for immunofluorescent stainings with polyclonal rabbit anti-Iba1 (1:500; Wako D19–19741) or rat anti-F4/80 (1:500; Serotec, Oxford, UK). For detection, HRP-labeled secondary antibodies (1:200) and fluorescent labeling with a cyanine 2 (FITC) TSA kit (Perkin Elmer Life Sciences, Boston, USA) were used. Cell nuclei were labeled with Vectashield Antifade Mounting Medium with DAPI (Vector Laboratories, United Kingdom). Images were acquired with a motorized inverted IX-81 microscope connected to a CCD-FV2T digital camera (Olympus, Aartselaar, Belgium) and processed with LSM Image Browser software (Zeiss, Germany).

Microglial cell numbers were counted around the sagittal midline and coronal plane at the height of brainstem and visual cortex. Within one plane (× 20 magnification), only Iba1-positive cells that (1) had fully co-localized with DAPI-positive nuclei, (2) had a clear cell soma, and (3) had at least two clear protrusions were counted in different regions of the brain. Microglial number per frame was corrected for surface area. Three to five different pictures per brain region per mouse were taken. The number of microglia was counted, and the average of all pictures per brain region was used to quantify the number of microglial cells per brain region (*n* = 4–6/group).

For the facial nerve axotomy model, brains were, after transcardial perfusion (described above), incubated in 30% sucrose in phosphate-buffered saline (PBS) at 4 °C until fully submerged. All tissues were protected from light. Samples were embedded in OCT (Tissue-Tek) for frozen sectioning on a cryostat (Leica). Cryosections from brains with unilateral lesions were prepared on slides and kept at − 20 °C until use. Tissues were rehydrated or permeabilized in blocking solution (0.1% Triton-X 100, 5% bovine albumin, normal goat serum, and PBS) for 1 h at room temperature and then incubated overnight at 4 °C with primary antibody diluted in 5% serum and PBS. The following primary antibodies were used: polyclonal rabbit anti-Iba1 (1:500; Wako 019-19,741) and rat anti-F4/80 (1:500; Serotec, Oxford, UK). Extensive wash steps were performed with PBS, and sections were incubated with goat anti-rabbit secondary antibody conjugated to Alexa Fluor 488 (1:1000; Life Technologies A32731) for 1 h at room temperature. Microglia responses were analyzed 1 day and 5 days post-axotomy. Iba1^+^ fluorescence intensity and numbers of microglia cells per area were measured in ipsi- and contralateral sides, and the ratio of ipsilateral side relative to their respective contralateral side is shown for both genotypes (*Cx3cr1-Mfp2*^*−/*−^ and control mice).

### Microglia isolation and cell culture

Microglial cells were isolated from control and *Mfp2*^*−/−*^ pups at postnatal day 8 (P8) using magnetic-activated cell sorting (MACS) according to the manufacturer’s instructions (Miltenyi Biotec). Cells were plated in 12-well plates in a Macrophage Serum Free Medium (Thermo Fisher) and stimulated for 24 h with 50 ng/ml IL1β and 20 ng/ml IFNγ or 50 ng/ml IL-4 (all from R and D) to induce a pro- and anti-inflammatory phenotype, respectively.

For confirming recombination of the *Mfp2* gene, microglia were isolated from 11-month-old control and *Cx3cr1-Mfp2*^*−/−*^ mice. Mice were anesthetized with a mix of Domitor (1 mg/kg) and Nimatek (75 mg/kg) and perfused with approximately 20 ml ice-cold HBSS (without calcium and magnesium). Subsequently, brains were removed and dissociated to a single cell suspension using the Neural Tissue Dissociation Kit (P) according to the manufacturer’s instructions for the automated dissociation using the gentleMACS Dissociator (Miltenyi Biotec). Next, myelin was removed by 22% Percoll gradient and the cell suspension was further processed for microglia separation. Microglia (positive fraction) were separated from other brain cells (negative fraction) by MACS following the manufacturer’s instructions using CD11b MicroBeads (Miltenyi Biotec). Both the positive and negative fractions were further processed for qRT-PCR.

### Real-time quantitative PCR

Total RNA was isolated from snap-frozen brain tissue using Trizol reagent (Invitrogen, California, USA) or from isolated or cultured microglia by using the PureLink RNA Mini Kit, both according to the manufacturer’s protocol. Subsequently, cDNA was generated from a 1-μg RNA using the QuantiTect Reverse Transcription Kit (QIAGEN, Venlo, The Netherlands). For real-time PCR, an ABI PRISM 7500 Real-Time PCR instrument (Applied Biosystems, Lennik, Belgium) was used. Primers and probes were ordered from Applied Biosystems as premade Taqman Gene Expression Assays (Il1b, Mm011336189_m1; Cx3cr1, Mm0262011_s1; Tgfbr1, Mm00436964_m1; arginase 1, Mm00475991_m1; Mrc1, Mm00485148_m1) and used as previously described [27]. Alternatively, the following genes were tested in triplicate using the PowerUp SYBR Green Master Mix (Thermo Fisher) with primers ordered from Integrated DNA Technologies (Leuven, Belgium): cd200, Mm.PT.58.33215550; Cx3cl1, Mm.PT.58.8767901; Csf1r, Mm.PT.58.12811749; Csf1, Mm.PT.58.11661276; Cxcl1, Mm.PT.58.8767901; Il34, Mm.PT.58.32379406; Fizz-1, Mm.PT.58.43062398; Il4, Mm.PT.58.7882098; Ym-1, Mm.PT.58.33370435; Tlr2, Mm.PT.58.45820113; Tspo, Mm.PT.58.43313736; F4/80 or Emr1, Mm.PT.58.11087779; Tmem119, Mm.PT.58.6766267; P2ry12, Mm.PT.58.43542033; and Mfp2, Mm.PT.58.16985875. The data were analyzed using the 2-ΔΔCT method [28]. The relative expression levels of the target genes were calculated as a ratio to the housekeeping gene β-actin except for the facial nerve injury experiment for which adaptor-related protein complex 3, delta 1 subunit (*Ap3d1*), F-box protein 38 (*Fbxo38*), and MON2 homolog (*Mon2*) were used that remain unaltered after neuronal injury [25]. The following primers were used: Ap3d1 (forward, 5′-CAAGGGCAGTATCGACCGC-3′; reverse, 5′-GATCTCGTCAATGCACTGGGA- 3′), Mon2 (forward, 5′-CTACAGTCCGACAG GTCGTGA-3′; reverse, 5′-CGGCACTGGAGGTTCTATATCTC-3′), and Fbxo38 (forward, 5′-ATGGGACCACGAAAG AAAAGTG-3′; reverse, 5′-TAGCTTCCGAGAGAGGCATTC-3′).

### MFP2 activity measurements

The forebrain of 11-month-old mice was homogenized in 5 mM MOPS pH 7.2, 1 mM EDTA, 250 mM sucrose, and 0.1% (*v*/*v*) ethanol. After the appropriate dilution of the samples, the dehydratase activity of MFP2 was measured as previously described [29] with 3S-hydroxy-3-phenylproprionyl-CoA as a substrate, except that Thesit was increased to 0.05% (*w*/*v*).

### Statistical analysis

All data except some behavioral tests (see below) were analyzed with GraphPad Prism software (version 5.0 and 6.0, San Diego, CA). Statistical analyses were carried out using unpaired and paired, two-sided Student’s *t* test, one-way ANOVA, two-way ANOVA, or two-way repeated measure (RM) ANOVA followed by the Bonferroni post hoc test. Data are shown as mean ± standard error of the mean (SEM), and statistical significance was set at *p* < 0.05. SPSS Statistics software was used for three-way ANOVA and three-way RM ANOVA.

#### BAEP test

Two-way ANOVA with genotype and (inter)peak as sources of variation was used to evaluate neuronal transmission in *Cx3cr1-Mfp2*^*−/−*^ mice. The Holm-Sidak and Bonferroni methods were used for multiple comparisons. Unpaired *t* test was used to evaluate the peak amplitudes.

#### OF and PA test

Independent sample *t* test and Mann-Whitney *U* test were used to compare performance between different genotypes.

## Results

### Generation of microglia/monocyte-specific *Cx3cr1*-*Mfp2*^*−/−*^ mice

In order to investigate whether inactivation of peroxisomal β-oxidation in microglia impacts on microglial behavior and may contribute to the neuropathology that we observed in *Mfp2*^*−/−*^ mice, we generated a microglia/monocyte-specific knockout, by crossbreeding *Cx3cr1*-*Cre* mice [20] with floxed *Mfp2* mice [2]. First, we analyzed whether insertion of the *Cre*-recombinase gene in the genome and haploinsufficiency of CX3CR1 did not negatively influence microglial behavior in the brain. Microglia were investigated in the brain of both *Cre*-positive and *Cre*-negative control mice at 5, 8, and 12 months of age. No differences in Iba1^+^ cells regarding morphology and cell numbers were observed in the brain of *Cre*-positive (*Cre Mfp2*^*Wt/LoxP*^) versus *Cre*-negative (*Mfp2*^*Wt/LoxP*^) control mice (Additional file 1: Figure S1). There were no signs of microglial reactivity, and the F4/80 marker was absent in both *Cre*-positive and *Cre*-negative control mice at all ages (data not shown). In addition, no differences in GFAP expression were observed in mice of both genotypes indicating that insertion of *Cre* did not influence astroglia (data not shown). Therefore, we used both *Cre*-positive and *Cre*-negative mice as control animals. Because reliable antibodies for immunohistochemical detection of MFP2 are not available [30], we confirmed the recombination of *Mfp2* in microglia of *Cx3cr1-Mfp2*^*−/−*^ mice using transcript analysis on MACS-isolated microglia. The selectivity of *Mfp2* recombination was further confirmed by the normal expression of MFP2 in the non-microglia fraction and by the fact that the activity of MFP2 was not significantly reduced in whole brain homogenates (Additional file 2: Figure S2). *Cx3cr1*-*Mfp2*^*−/−*^ mice were indistinguishable from their control littermates during the first year of life, were fertile and survived past the age of 14 months.

### Development of microglial proliferation and morphological transformation in the brain of *Cx3cr1-Mfp2*^*−/−*^ mice

**Fig. 1:**
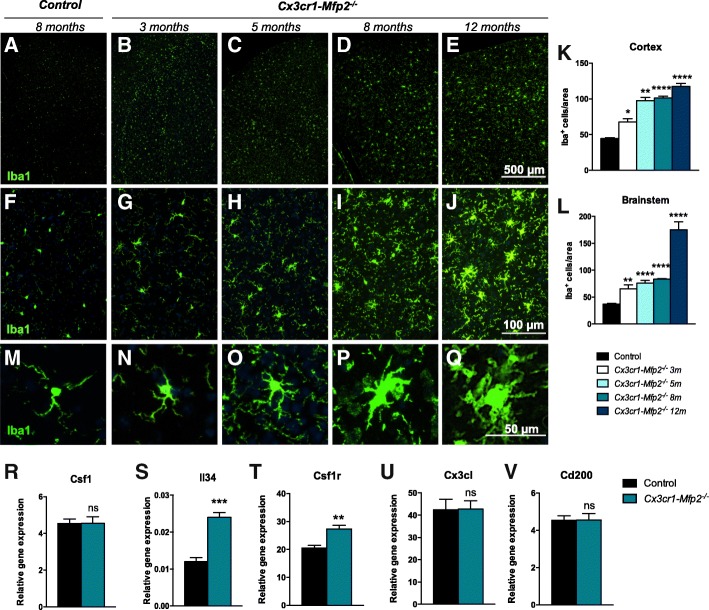
Development of microgliosis in *Cx3cr1-Mfp2*^*−/−*^ brain. **a**–**e** Overview pictures of Iba1^+^ cells (green) in visual cortex of control (**a**) and *Cx3cr1-Mfp2*^*−/−*^ mice (**b**–**e**) at the indicated ages and at higher magnification (**f**–**j**). **k**, **l** Quantification of microglial cells in the cortex (visual and motor cortex) and the brainstem of *Cx3cr1-Mfp2*^*−/−*^ mice (*n* = 4 mice/age) compared to control mice. Data of control mice across different ages (3, 5, 8, 12 months) is combined (*n* = 16 mice). **m**–**q** Gradual morphological transformation of microglia in *Cx3cr1-Mfp2*^*−/−*^ brain at the same ages. **r**–**v** Gene expression analysis by qRT-PCR of markers related to the proliferation of microglia in 8-month-old mice. *Cx3cr1-Mfp2*^*−/−*^ mice compared to control: **p < 0.05, ***p < 0.001, ****p < 0.0001. ns, not significant. Error bars indicate SEM. m, months. *n* = 4 mice/group. Representative pictures are shown

Constitutive *Mfp2*^*−/−*^ mice develop severe and extensive microgliosis from 6 weeks of age that progressively increases. In contrast, *Nestin-Mfp2*^*−/−*^ mice only develop microgliosis from 12 to 17 weeks of age, which is never as extensive as in constitutive *Mfp2*^*−/−*^ mice [11], suggesting that intrinsic loss of MFP2 from microglia might be involved in the development of microgliosis. In order to address this, we analyzed cell numbers and morphology of microglia in *Cx3cr1-Mfp2*^*−/−*^ mice at different ages. IHC analysis and quantification of the microglial marker Iba1 revealed that numbers of microglial cells progressively increased from the age of 3 months in all brain regions, both in gray and white matter, of *Cx3cr1-Mfp2*^*−/−*^ mice compared to age-matched control mice (Fig. 1a–l). In the control mice, microglial cell numbers were similar across all ages that were investigated, so the data were combined (Fig. 1k, l). The proliferation of microglial cells was associated with progressive morphological transformation (Fig. 1m–q). At 3 months of age, several microglia in the *Cx3cr1-Mfp2*^*−/−*^ brain developed thicker and shorter protrusions and a mildly enlarged cell soma (Fig. 1n). These hypertrophic features became gradually more pronounced with age (Fig. 1o–q).

Microglial proliferation is typically induced by the cytokines colony-stimulating factor 1 (CSF1) or interleukin-34 (IL-34) that are ligands of the CSF1R. We analyzed whether the gene expression of these cytokines was changed in the brain of *Cx3cr1-Mfp2*^*−/−*^ mice by qPCR analysis. Transcript levels of *Csf1* were not altered (Fig. 1r), but transcript levels of *Il34* and *Csf1r* were induced (Fig. 1s, t) in 8-month-old *Cx3cr1-Mfp2*^*−/−*^ mice in comparison to age-matched control mice.

In the healthy brain, neurons chronically restrain microglia in order to maintain their surveilling state and prevent microglial proliferation. To investigate whether the microglial proliferation was related to the loss of neuronal restraint signals, we analyzed pivotal markers in the brains of *Cx3cr1-Mfp2*^*−/−*^ mice. We found that transcript levels of *Cx3cl* (*fractalkine*) (Fig. 1u) and *Cd200* (Fig. 1v) were equal to those in age-matched control brains. Taken together, the deletion of the peroxisomal β-oxidation enzyme MFP2 from microglia induces progressive morphological changes and IL-34-driven proliferation, typical features of microglial activation. In comparison to constitutive *Mfp2*^*−/−*^ mice, the microgliosis is less pronounced and delayed in *Cx3cr1-Mfp2*^*−/−*^ brain [10, 11].

### Microglia in *Cx3cr1-Mfp2*^*−/−*^ mice are inflammatory activated and adopt a pro-inflammatory state at later stages

**Fig. 2:**
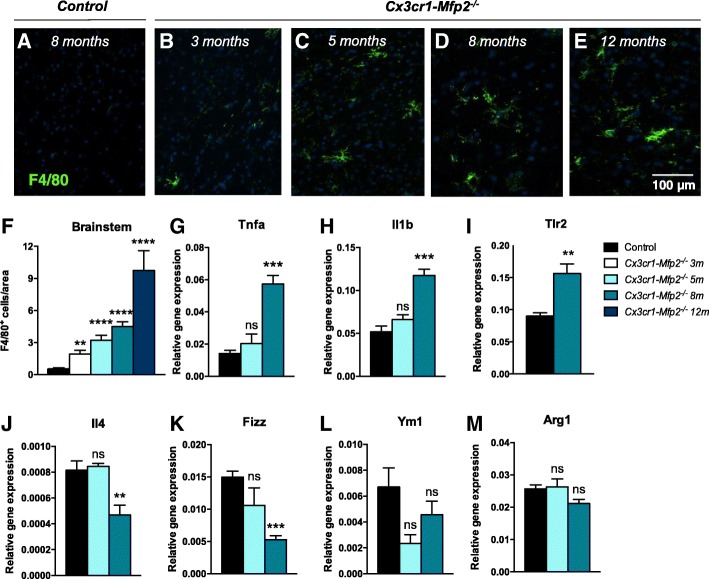
Inflammatory characteristics of *Cx3cr1-Mfp2*^*−/−*^ brain. **a**–**e** F4/80^+^ cells (green) are absent in the brains of control mice (**a**), but F4/80^+^ cells increase with age in *Cx3cr1-Mfp2*^*−/−*^ brains (**b**–**e**). Cell nuclei are stained blue with DAPI. Representative pictures of the brainstem are shown. **f** Quantification of F4/80^+^ cells in the brainstem of *Cx3cr1-Mfp2*^*−/*−^ mice (*n* = 4 mice/age) compared to control mice. Data of control mice across different ages (3, 5, 8, 12 months) is combined (*n* = 16). **g**–**i** Transcript levels of pro-inflammatory markers in *Cx3cr1-Mfp2*^−/−^ brain at 5 and 8 months of age. **j**–**m** Transcript levels of anti-inflammatory markers in *Cx3cr1-Mfp2*^*−/−*^ brain at 5 and 8 months of age. *Cx3cr1-Mfp2*^*−/−*^ mice compared to control: **p < 0.01, ***p < 0.001, ****p < .0001. ns, not significant. Error bars indicate SEM. *n* = 4–8 mice/group; m, months

The microglia/macrophage marker F4/80 becomes upregulated on the microglial membrane when microglia get activated in response to the disruption of CNS homeostasis, such as neuronal injury, aging, or infectious pathogens in the brain parenchyma. Numbers of activated F4/80^+^ microglia increased with age in *Cx3cr1-Mfp2*^*−/−*^ mice during the first year of life (Fig. 2a–f), whereas F4/80^+^ cells were absent in the control mice at all ages (Fig. 2a). Microglial activation is accompanied with the induction of inflammatory markers in pathological conditions. The transcript levels of the pro-inflammatory markers *Tnfa*, *Il1b*, and *Tlr2* were not significantly changed at 5 months of age but were induced at 8 months of age in *Cx3cr1-Mfp2*^*−/−*^ mice (Fig. 2g–i). We assessed whether induction of pro-inflammatory markers was associated with the downregulation of anti-inflammatory markers. Transcripts of *Il4* (Fig. 2j) and *Fizz* (Fig. 2k) were downregulated at 8 months, but not at 5 months of age. Transcript levels of *Ym1* (Fig. 2l) and *Arginase-1* (*Arg1*) (Fig. 2m) were not significantly decreased. Together, this indicates that *Mfp2*^*−/−*^ microglia in *Cx3cr1-Mfp2*^*−/−*^ mice adopt an activated pro-inflammatory phenotype.

### Normal responses of *Mfp2*^*−/−*^ microglia to pro- and anti-inflammatory challenges in vivo and in vitro

**Fig. 3:**
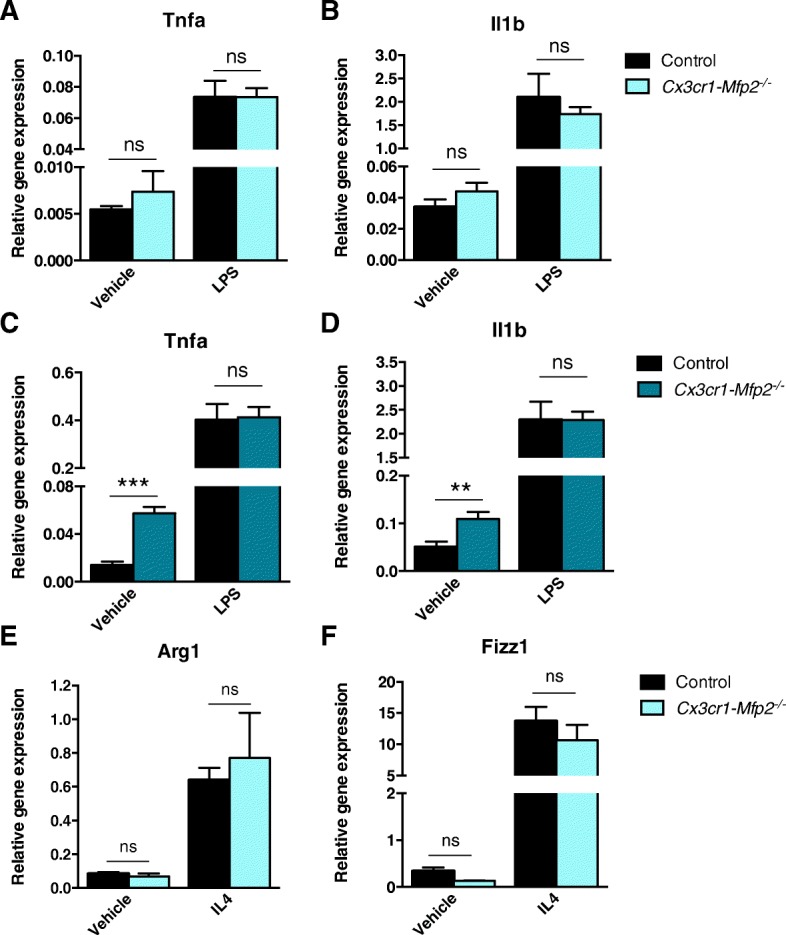
*Cx3cr1-Mfp2*^*−/−*^mice respond adequately to pro- and anti-inflammatory challenges. **a**–**d** Five-month-old (**a**, **b**) and 8-month-old (**c**, **d**) *Cx3cr1-Mfp2*^*−/−*^ and control mice were challenged with i.p. LPS or vehicle, and the brainstem was analyzed after 6 h for transcript levels of pro-inflammatory markers. *n* = 4–6 mice/group. **e**, **f** Five-month-old *Cx3cr1-Mfp2*^*−/−*^ and control mice were challenged with i.c.v. IL-4 or vehicle, and the frontal cortex contralateral to the injection site was analyzed after 20 h for transcript levels of anti-inflammatory markers. *n* = 3 mice/group. *Cx3cr1-Mfp2*^*−/−*^ mice compared to control: **p < 0.05, ***p < 0.001. ns, not significant. Error bars indicate SEM

We subsequently assessed how microglia in *Cx3cr1-Mfp2*^*−/−*^ mice respond to pro- and anti-inflammatory stimuli. LPS was systemically administered to 5-month-old (Fig. 3a, b) and 8-month-old (Fig. 3c, d) mice, and transcript levels of pro-inflammatory markers were monitored. Expression of *Tnfa* (Fig. 3a, c) and *Il1b* (Fig. 3b, d) were similarly induced in the *Cx3cr1-Mfp2*^*−/−*^ and control mice after LPS injection at both ages, indicating that microglia in *Cx3cr1-Mfp2*^*−/−*^ mice are not primed. Subsequently, we investigated responses to an anti-inflammatory stimulus by performing i.c.v. injections of IL-4 in the *Cx3cr1-Mfp2*^*−/−*^ and control mice at 5 months of age. Both the *Cx3cr1-Mfp2*^*−/−*^ and control mice exhibited an anti-inflammatory brain environment post-injection, evident by increased expression of anti-inflammatory markers (*Arg1* and *Fizz1*) (Fig. 3e, f) and unaltered expression of pro-inflammatory markers (*Tnf, Il1b*, and *Tlr2*, data not shown). The data demonstrate normal responsiveness of *Mfp2*^*−/−*^ microglia to an anti-inflammatory challenge as anti-inflammatory markers in *Cx3cr1-Mfp2*^*−/−*^ mice were elevated to the same extent as in control mice.

Finally, we tested whether cultured *Mfp2*^*−/−*^ microglia derived from P8 *Mfp2*^*−/−*^ mice react normally to cytokine exposure. Under basal conditions, no differences in expression levels of pro- or anti-inflammatory markers were observed (Additional file 3: Figure S3). Likewise, when challenged with a pro- or anti-inflammatory stimulus, *Mfp2*^*−/−*^ microglia responded similarly as compared to the control microglia (Additional file 3: Figure S3). Taken together, our results show that *Mfp2*^*−/−*^ microglia are not primed and respond normally to pro- and anti-inflammatory stimuli.

### *Mfp2*^*−/−*^ microglia in *Cx3cr1-Mfp2*^*−/−*^ mice exhibit a normal response to neuronal injury

**Fig. 4:**
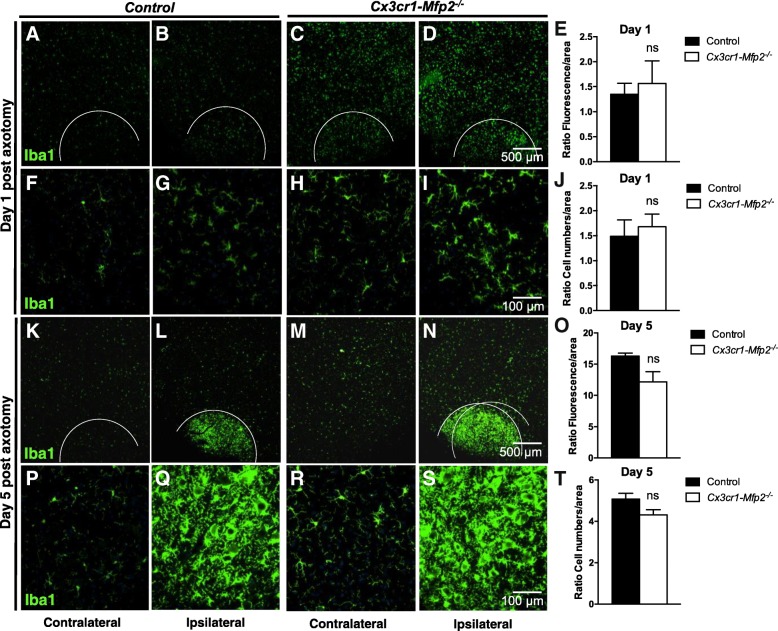
Microglia in *Cx3cr1-Mfp2*^*−/−*^ brain react normally to nerve injury. **a**–**t** Facial nerve was axotomized at the left side (ipsilateral) of the brain, whereas the right facial nerve remained intact (contralateral) in 3-month-old *Cx3cr1-Mfp2*^*−/−*^ and control mice (*n* = 3–5 mice/group). Microgliosis was analyzed in facial motor nucleus (FMN) in the brainstem at 1 day (**a**–**j**) and 5 days post-axotomy. **a**–**d**, **k**–**n** Overview pictures of the brainstem and FMN regions (marked by a white line) in control and *Cx3cr1-Mfp2*^*−/−*^ mice are shown at 1 day (**a**–**d**) and 5 days (**k**–**n**) post-axotomy. **f**–**i**, **p**–**s** Magnifications of FMN in control and *Cx3cr1-Mfp2*^*−/−*^ mice at 1 day (**f**–**i**) and 5 days (**k**–**n**) post-axotomy. Both ipsilateral and contralateral sides are shown. **e**, **o** Quantification of Iba1^+^ fluorescence intensity in FMN in ipsilateral sides relative to their respective contralateral sides at 1 day (**e**) and 5 days (**o**) post-axotomy. **j**, **t** Quantification of Iba1^+^ cell numbers in FMN in ipsilateral relative to their respective contralateral sides at 1 day (**j**) and 5 days (**t**) post-axotomy. Representative pictures are shown. *Cx3cr1-Mfp2*^*−/−*^ mice compared to control: * *p* < 0.05. ns, not significant. Error bars indicate SEM

In order to elucidate how *Mfp2*^*−/−*^ microglia respond to neuronal injury, we induced unilateral transection of the facial nerve (FN) in 3-month-old *Cx3cr1-Mfp2*^*−/−*^ and control mice. The contralateral side remained intact and was considered as the control side. Facial nerve axotomy provokes a local microglial response in the facial motor nucleus (FMN) in the brainstem [31–33] which reaches a maximum at 5 days post-injury [25]. The microglial response in FMN was analyzed at day 1 (Fig. 4a–j) and at day 5 post-axotomy (Fig. 4k–t). As microglia are activated and cell numbers increased in intact 3-month-old *Cx3cr1-Mfp2*^*−/−*^ relative to control brains (Fig. 1), Iba1^+^ fluorescence intensity and cell numbers in ipsilateral FMNs were measured relative to their contralateral FMNs (ratio) in both genotypes. Iba1+ intensity and cell numbers were similarly increased in *Cx3cr1-Mfp2*^*−/−*^ and control mice after facial nerve axotomy at day 1 (Fig. 4e, j) and at day 5 post-axotomy (Fig. 4o, t). At day 1 post-injury, fold change measurements of Iba1^+^ fluorescence in ipsilateral FMN relative to contralateral FMN indicated that microglial response to acute neurodegeneration is similar in *Cx3cr1-Mfp2*^*−/−*^ (1.6-fold) and control (1.4-fold) mice, in parallel with similarly increased microglial numbers in ipsilateral FMN in control (1.5-fold) and *Cx3cr1-Mfp2*^*−/−*^ (1.7-fold) mice. This indicates that *Mfp2*^*−/−*^ microglia respond normally to neuronal injury immediately after lesion was generated.

At 5 days post-axotomy, an extensive microglial response was generated in the ipsilateral FMN of both control (Fig. 4l, q) and *Cx3cr1-Mfp2*^*−/−*^ (Fig. 4n, s) mice. We found that there was no significant difference in the induction of microglial response in the ipsilateral FMN of control (16-fold) and *Cx3cr1-Mfp2*^*−/−*^ (13-fold) mice (Fig. 4o). Accordingly, microglial numbers are equally increased in the ipsilateral FMN of control (5.1-fold) and *Cx3cr1-Mfp2*^*−/−*^ (4.3-fold) mice (Fig. 4t). Taken together, the neuronal injury did not provoke an exaggerated or diminished inflammatory response in *Mfp2*^*−/−*^ microglia during the acute post-injury period. Accordingly, no differences were observed in F4/80^+^ microglial cells and GFAP^+^ astroglial cells in the ipsilateral FMNs of *Cx3cr1-Mfp2*^*−/−*^ and control brain at day 1 and 5 post-axotomy (data not shown).

**Fig. 5:**
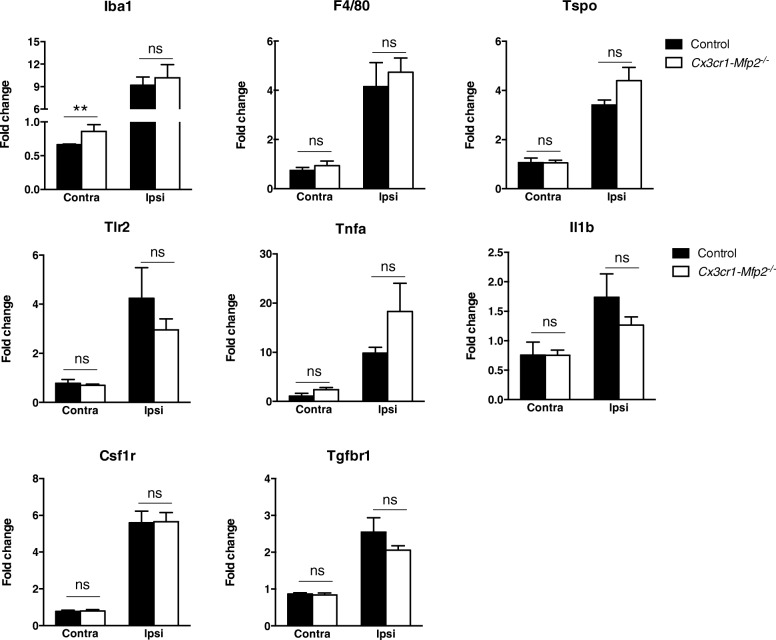
Microglia in *Cx3cr1-Mfp2*^*−/−*^ brain adopt a normal inflammatory phenotype after nerve injury. Facial nerve was axotomized at the left side (ipsilateral) of the brain, whereas the right facial nerve remained intact in 3-month-old *Cx3cr1-Mfp2*^*−/−*^ and control. Transcript levels of inflammatory and microglial markers were analyzed in facial nuclei at 5 days post-axotomy. The expression of all markers significantly increased at the axotomized ipsilateral side (ipsi) of the brain versus intact contralateral side (contra) in both *Cx3cr1-Mfp2*^*−/−*^ and control mice (significance levels not shown). There were no differences between genotypes at the affected ipsilateral side. *Cx3cr1-Mfp2*^*−/−*^ mice compared to control: *p < 0.05, **p < 0.01, ****p < 0.0001. ns, not significant. *n* = 5 mice/group. Error bars indicate SEM

To assess the inflammatory profile of proliferated microglia, FMN at contra- and ipsilateral sides was isolated at 5 days post-axotomy from *Cx3cr1-Mfp2*^*−/−*^ and control brain, and transcript levels of several inflammatory molecules were measured by qPCR analysis. In basal conditions, represented by the contralateral side, only the transcript levels of *Iba1* were significantly induced in *Cx3cr1-Mfp2*^*−/−*^ compared to control mice. At the axotomized ipsilateral side, the expression of inflammatory markers being the microglial marker *Iba1* (Fig. 5a); activation marker *F4/80* (Fig. 5b); neuroinflammatory marker *Tspo* (Fig. 5c); pro-inflammatory markers *Tlr2*, *Tnfa*, and *Il1b* (Fig. 5d, e, f); proliferative microglial receptor *Csf1r* (Fig. 5g); and homeostatic microglial marker *Tgfbr1* (Fig. 5h) were similarly induced versus the intact contralateral FMNs in both genotypes. These results show that *Mfp2*^*−/−*^ microglia in the *Cx3cr1-Mfp2*^*−/−*^ brain exhibit a normal inflammatory reaction to acute neuronal injury.

### *Cx3cr1-Mfp2*^*−/−*^ mice exhibit intact neuronal functioning and a normal clinical phenotype and cognition

**Fig. 6:**
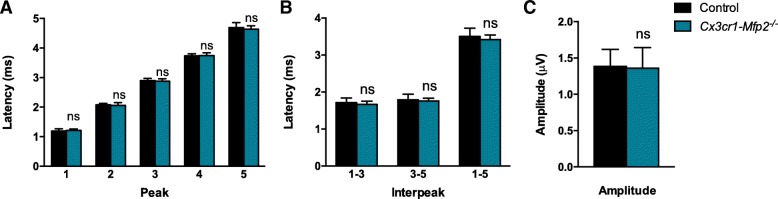
Intact neuronal functioning in *Cx3cr1-Mfp2*^*−/−*^ brain. **a**–**c** BAEP test shows normal brainstem responses in *Cx3cr1-Mfp2*^*−/−*^ versus control mice at 8 months of age. **a** Mean peak latencies show that all peaks assigned to specific brainstem regions (peak 2–4) and thalamus/cortex regions (peak 5) show normal latencies of the auditory signal in *Cx3cr1-Mfp2*^*−/−*^ mice. **b** Normal brain responses to the auditory stimulus were shown by similar interpeak latencies in *Cx3cr1-Mfp2*^*−/−*^ and control mice. **c** Peak amplitudes are normal in *Cx3cr1-Mfp2*^*−/−*^ versus control mice. *n* = 5–9 mice/group. ns, not significant. Error bars indicate SEM

We found previously that neuronal transmission of auditory signals in the brain was severely delayed and peak amplitudes were reduced in constitutive *Mfp2*^*−/−*^ mice after evoking auditory potentials in the brainstem (BAEPs). Decreased neuronal transmission progressed in parallel with aggravating neuroinflammation [11]. In contrast, there was only a minor delay in transmission and normal peak responses of evoked auditory signals in *Nestin-Mfp2*^*−/−*^ mice [11]. In order to reveal whether a pro-inflammatory state of microglia in *Cx3cr1-Mfp2*^*−/−*^ mice affects auditory brainstem responses, BAEPs were analyzed in 8-month-old *Cx3cr1-Mfp2*^*−/−*^ and control mice. Peak latencies show the time when the auditory signal evokes a response in a specific auditory nucleus in the brain [34]. Mean peak and interpeak latencies were similar in *Cx3cr1-Mfp2*^*−/−*^ and control mice (Fig. 6a,b), and peak amplitudes are normal in *Cx3cr1-Mfp2*^*−/−*^ versus control mice (Fig. 6c). These results demonstrate that neuronal signal transmission and amplitudes of brainstem responses are intact in *Cx3cr1-Mfp2*^*−/−*^ mice.

**Fig. 7:**
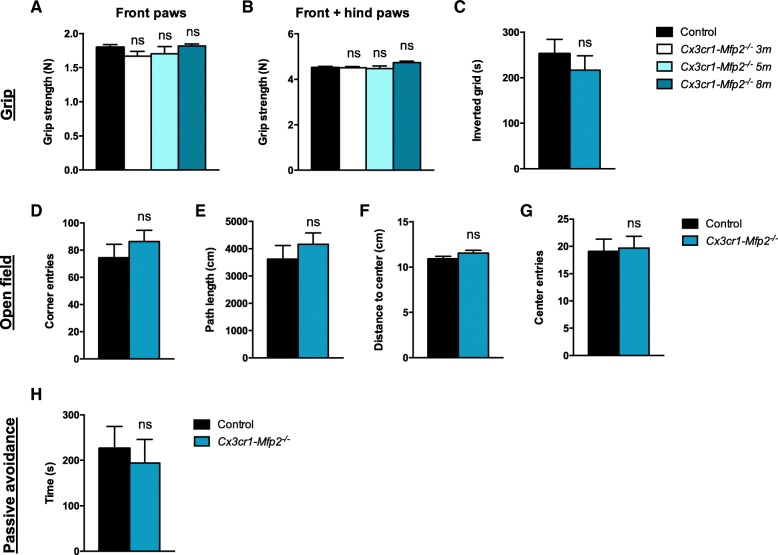
*Cx3cr1-Mfp2*^*−/−*^mice show a normal clinical phenotype. **a**, **b** Normal grip strength in front paws (**a**) and all paws together (**b**) in 3-, 5-, and 8-month-old *Cx3cr1-Mfp2*^*−/−*^ mice compared to control mice. **c** Inverted grid test shows similar performances regarding grip strength and coordination in 8-month-old *Cx3cr1-Mfp2*^*−/−*^ mice and control mice. **d**, **e** Eight-month-old *Cx3cr1-Mfp2*^*−/−*^ mice show normal locomotor activity as the number of entries in the corners (**d**) and total path length (**e**) in an open field environment is similar to control mice. **f**, **g** Eight-months-old *Cx3cr1-Mfp2*^*−/−*^ mice exhibit normal exploratory behavior as the mean distance to the center (**f**) and number of entries in the center (**g**) are similar to control mice in an open field environment. **h** Fear-conditioned memory was assessed by means of the passive avoidance test. *Cx3cr1-Mfp2*^*−/−*^ show a similar delay to traverse to the dark compartment as control mice indicative of a normal cognition. ns, not significant. Error bars indicate SEM. m, months. *n* = 5–12 mice/group

Whereas constitutive *Mfp2*^*−/−*^ mice succumb before the age of 6 months and show severely impaired locomotor activity, exploration, and fear conditioning as an index of cognition at early age, the neural-specific *Nestin*-*Mfp2*^*−/−*^ mice survive up to 1 year and clinical impairments were delayed and less pronounced at the end stage of disease [11]. In order to investigate whether microglia-restricted loss of MFP2 affects grip strength, motor abilities, and behavior, the same tests were performed on *Cx3cr1*-*Mfp2*^*−/−*^ mice at the age of 8 months when inflammatory activation is manifested. We found that grip strength of front paws (Fig. 7a) and all paws together (Fig. 7b) remains intact in *Cx3cr1*-*Mfp2*^*−/−*^ mice. Grip strength and coordination on an inverted grid did not differ in *Cx3cr1*-*Mfp2*^*−/−*^ mice and age-matched control mice (Fig. 7c). *Cx3cr1-Mfp2*^*−/−*^ mice displayed normal locomotor activity in an open field environment as they display a similar amount of corner entries (Fig. 7d) and similar path length (Fig. 7e) as compared to control mice. *Cx3cr1-Mfp2*^*−/−*^ mice exhibited a normal explorative behavior as the mean distance to the center (Fig. 7f), number of center entries (Fig. 7g), and time in the center (not shown) were similar to the control mice. Fear-conditioned memory was analyzed by passive avoidance test. There was no significant difference in time when *Cx3cr1-Mfp2*^*−/−*^ and control mice enter into the dark room during training (data not shown). During the test phase, *Cx3cr1-Mfp2*^*−/−*^ and control mice showed a similar delay in time to enter the dark room (Fig. 7h), demonstrating that the 8-month-old *Cx3cr1-Mfp2*^*−/−*^ mice have a normal cognition. Taken together, we show that *Mfp2*^*−/−*^ microglia in a genetically intact brain environment do not induce abnormalities in motor function, explorative behavior, and cognition before 8 months of age.

## Discussion

In this study, we investigated the impact of deletion of the pivotal peroxisomal β-oxidation enzyme MFP2 from microglia on immune response and neural functioning. Previous data demonstrated that global loss of MFP2 elicited an early-onset and extensive microgliosis in the CNS that was accompanied by quick deterioration of the mice [2, 10]. Here, we established that *Mfp2*^*−/−*^ microglia in a genetically intact CNS environment adopt an inflammatory activated and proliferative state. Despite the pro-inflammatory microglial state, *Cx3cr1-Mfp2*^*−/−*^ mice exhibited normal clinical performance and cognition. In addition, we found that acute inflammatory and neuronal injury provoked normal responses of *Mfp2*^*−/−*^ microglia in *Cx3cr1-Mfp2*^*−/−*^ mice during the post-injury period. Our data strongly suggest that MFP2 deficiency in microglia causes intrinsic pro-inflammatory deregulation, which is not harmful for neuronal function, motor function, and cognition in mice during their first year of life.

Histological examination of the *Cx3cr1*-*Mfp2*^*−/−*^ brain demonstrated progressive development of microgliosis in the absence of neuronal degeneration. Our results indicate that microglial proliferation is stimulated by IL-34 rather than by CSF1 signaling. The CSF1R is ligated by both CSF1 and IL-34 which play redundant roles in developing and adult brain [35]. It was demonstrated that IL-34 binding on CSF1R promotes maintenance and proliferation of microglia in the brain [36–39]. Although both IL-34 and CSF1 may induce microglial proliferation upon neurodegeneration, IL-34 has a stronger proliferation-inducing capacity and higher expression in the brain compared to CSF1 [35, 40]. Despite identical signaling pathways induced by CSF1 and IL-34 downstream CSF1R, a recent study found that IL-34-stimulated monocytes produce different cytokines/chemokines in an inflammatory context and exhibit a distinct polarization potential compared to CSF1-derived macrophages [41]. There is indeed increasing evidence that IL-34 expression is upregulated in pathological conditions and plays important roles in autoimmune disorders, infections, and inflammatory conditions [42]. Previous results showed that *Il34* levels were also increased in constitutive *Mfp2*^*−/−*^ mice (eightfold) and neural-specific *Nestin-Mfp2*^*−/−*^ mice (fivefold) [11], whereas *Csf1* levels were unchanged in both mouse models at the end stage of disease. Further research is however necessary to elucidate the distinct biological profile and effects of both cytokines.

The proliferation of microglia in *Cx3cr1*-*Mfp2*^*−/−*^ mice was accompanied with morphological transformation characterized by a swollen cell soma and thicker and shorter protrusions, both typical features of microglial activation [15, 43]. A progressive expansion of F4/80^+^ cells in *Cx3cr1*-*Mfp2*^*−/−*^ brains verified that increasing numbers of *Mfp2*^*−/−*^ microglia are activated from at least 3 months of age. In contrast to *Mfp2*^*−/−*^ microglia in constitutive *Mfp2*^*−/−*^ mice, which adopt a mixed pro- and anti-inflammatory phenotype, microglia in *Cx3cr1*-*Mfp2*^*−/−*^ mice downregulate anti-inflammatory and induce pro-inflammatory cytokines from 8 months of age. This demonstrates that *Mfp2*^*−/−*^ microglia in an intact CNS environment adopt a pro-inflammatory state several months after microglial proliferation and activation were initiated. These observations are in line with the pro-inflammatory profile of BV2 microglia in which another peroxisomal β-oxidation enzyme, ACOX1, was inactivated [44].

It should be noted that transient *Cx3cr1* promoter activity has been detected in neurons during the development in some mouse models [45]. However, in *Cx3cr1*-*Mfp2*^*−/−*^ mice, we did not find evidence for the neuronal inactivation of MFP2 based on normal *Mfp2* transcripts in non-microglial cells and the preservation of MFP2 activity in whole brain homogenates of *Cx3cr1*-*Mfp2*^*−/−*^ mice. The latter is compatible with the fact that microglia only constitute approximately 10% of brain cells [46, 47] and that MFP2 expression in microglia is lower or similar to the more abundant cell types [48]. An additional argument supporting that the reactive microglial phenotype does not depend on the neuronal inactivation of MFP2 is the fact that neuronal deficits such as Purkinje cell degeneration were not observed in *Cx3cr1*-*Mfp2*^*−/−*^ mice, in contrast to constitutive *Mfp*2 knockouts [2]. Finally, microglial proliferation and transformation were not only seen in gray but also in white matter regions.

We also examined how *Mfp2*^*−/−*^ microglia respond to acute inflammatory stimuli or to neuronal injury at an early disease stage, before inflammatory polarization was established. *Mfp2*^*−/−*^ microglia show normal responses to acute inflammatory stimuli in vitro and in vivo. *Mfp2*^*−/−*^ microglia acquired anti-inflammatory properties in response to an IL-4 stimulus which were indistinguishable from control microglia. An acute systemic pro-inflammatory stimulus elicited a similar response in microglia in *Cx3cr1*-*Mfp2*^*−/−*^ compared to control mice, proving that microglia in *Cx3cr1*-*Mfp2*^*−/−*^ mice are not primed despite their inflammatory activated state in unstimulated conditions. In contrast, *Mfp2*^*−/−*^ microglia in *Mfp2*^*−/−*^ mice are primed in the absence of systemic inflammation, and neuronal transmission is severely disturbed, suggesting that dysfunctional microglia-neuron bidirectional communication might trigger microglial priming and rapid progression of disease in constitutive *Mfp2*^*−/−*^ mice [11]. Neuronal injury was induced by unilateral axotomy of the facial nerve of *Cx3cr1*-*Mfp2*^*−/−*^ mice. We found that microglial responses in the axotomized relative to the intact FMN in *Cx3cr1*-*Mfp2*^*−/−*^ and control brain were comparable. Similar expression levels of several pro- and anti-inflammatory molecules in the ipsilateral FMN of *Cx3cr1*-*Mfp2*^*−/−*^ and control mice confirmed that *Mfp2*^*−/−*^ microglia do not elicit an exaggerated response to nerve injury during the acute post-injury period. In conclusion, microglia in *Cx3cr1*-*Mfp2*^*−/−*^ mice respond properly to acute pro- and anti-inflammatory challenges and to neuronal injury.

Our previous study demonstrated clear differences in the neuropathology of constitutive *Mfp2*^*−/−*^ mice versus neural-specific *Nestin-Mfp2*^*−/−*^ mice that were paralleled by distinct microglial phenotypes, indicating that *Mfp2*^*−/−*^ microglia play a role in the severe neuropathology of *Mfp2*^*−/−*^ mice [11]. However, we found that selective deletion of MFP2 from microglia in the *Cx3cr1-Mfp2*^*−/−*^ brain did not affect grip strength, locomotor activity, explorative behavior, and fear conditioning as an index of cognitive function within the time frame wherein all *Mfp2*^*−/−*^ mice and *Nestin-Mfp2*^*−/−*^ mice have died from the disease [19]. Hence, the early-onset clinical and behavioral abnormalities in *Mfp2*^*−/−*^ mice can neither be assigned purely to neuronal deficits nor to intrinsic microglial pathology. Likewise, the BAEP test demonstrated that brainstem responses and peak amplitudes are normal in *Cx3cr1*-*Mfp2*^*−/−*^ mice in contrast to affected responses and amplitudes in *Mfp2*^*−/−*^ mice and to a lesser extent in *Nestin*-*Mfp2*^*−/−*^ mice [11]. In the healthy brain, neurons persistently restrain microglia in order to maintain their surveilling state. Neurons in danger downregulate these restraint signals and send out “help me” signals that trigger microglial activation in pathological situations [15, 49, 50]. In *Mfp2*^*−/−*^ brain, the impaired neuronal signaling in the BAEP test was associated with lowered expression of the neuronal restraint signals *Cx3cl* and *Cd200*. In accordance to intact neuronal functioning in *Cx3cr1*-*Mfp2*^*−/−*^ mice, the expression of these neuron-microglia signaling molecules was normal in *Cx3cr1*-*Mfp2*^*−/−*^ brain. This indicates that intrinsic microglial pathology by itself is not sufficient to cause early-onset dysfunctional neuronal transmission in *Mfp2*^*−/−*^ mice. Taking into account all data on microglial reactivity, neuronal functioning, and neuropathological features, we hypothesize that impaired functioning of the CNS and clinical deterioration in *Mfp2*^*−/−*^ deficiency occur through synergistic instability of distinct brain cell types in *Mfp2*^*−/−*^ mice.

## Conclusion

We demonstrated in this study that *Mfp2*^*−/−*^ microglia in a genetically intact brain environment intrinsically adopt a proliferative and modified inflammatory state. The mild pro-inflammatory phenotype acquired by *Mfp2*^*−/−*^ microglia does not give rise to neuronal dysfunction nor to abnormal clinical behavior. Nevertheless, we cannot exclude that microglia become neurotoxic at a later stage. Our data indicate that microglia can develop a chronically proliferative and pro-inflammatory phenotype through cell-autonomous dysfunction without affecting the CNS environment and murine clinical behavior.

## Additional files

Additional file 1: **Figure S1.** No microgliosis in both Cre-positive and Cre-negative control mice. (A-D) No differences in microglia number and shape are observed between Cre-positive (*Cre Mfp2*^*Wt/LoxP*^) and Cre-negative (*Mfp2*^*Wt/LoxP*^) control mice at 5 months of age (A-D) and 12 months of age (not shown). Representative pictures are shown. *n* = 3–5 mice/group. (PPTX 988 kb)

Additional file 2: **Figure S2.** Efficient and selective inactivation of MFP2 in microglia in *Cx3cr1-Mfp2*^*−/−*^ mice. (A) Microglia were isolated from 11-month-old control and *Cx3cr1-Mfp2*^*−/−*^ mice, and microglia purity was confirmed by the high expression of microglial markers (Tmem119 and P2ry12) in the positive (microglia) versus the negative fraction (neurons, astrocytes, and oligodendrocytes). Transcript expression of *Mfp2* was determined in control and *Cx3cr1-Mfp2*^*−/−*^ in both positive and negative fraction. Representative experiment out of two with similar results. (B) MFP2 activity in brain homogenates of control and *Cx3cr1-Mfp2*^*−/−*^ mice. *n* = 2–3 mice/group. Mean ± SD is shown. (PPTX 46 kb)

Additional file 3: **Figure S3.** Inflammatory properties of cultured Mfp2^−/−^ and control microglia. MACS-isolated microglia from P8 mice were kept either in basal conditions or polarized to a pro-inflammatory state (Il1β/IFNγ) or an anti-inflammatory state (IL4). Transcript expression of pro-inflammatory (Tnfa, iNOS, Cxcl1) and anti-inflammatory cytokines (Arg1, Fizz1, Ym1) were determined. Significance levels: Φ *p* < 0.05, ΦΦ *p* < 0.01, ΦΦΦΦ *p* < 0.0001; ns, not significant. *n* = 8–11 mice/group. (PPTX 59 kb)

## Abbreviations

BAEP: Brainstem auditory evoked potentials
CNS: Central nervous system
CSF1R: Colony-stimulating factor 1 receptor
FMN: Facial motor nucleus
i.c.v.: Intracerebroventricular
IHC: Immunohistochemistry
Il: Interleukin
LPS: Lipopolysaccharide
MFP2: Multifunctional protein 2
OF: Open field
PA: Passive avoidance
