# Supplementary material for: Microglia lacking a peroxisomal β-oxidation enzyme chronically alter their inflammatory profile without evoking neuronal and behavioral deficits
Source: J Neuroinflammation. 2019 Mar 13;16:61. doi: 10.1186/s12974-019-1442-3 (PMC6417251; doi:10.1186/s12974-019-1442-3)
Supplement: Supplementary file 1 — Figure S1. No microgliosis in both Cre-positive and Cre-negative control mice. (A-D) No differences in microglia number and shape are observed between Cre-positive (Cre Mfp2Wt/LoxP) and Cre-negative (Mfp2Wt/LoxP) control mice at 5 months of age (A-D) and 12 months of age (not shown). Representative pictures are shown. n = 3–5 mice/group. (PPTX 988 kb) [file 12974_2019_1442_MOESM1_ESM.pptx]

## Slide 1
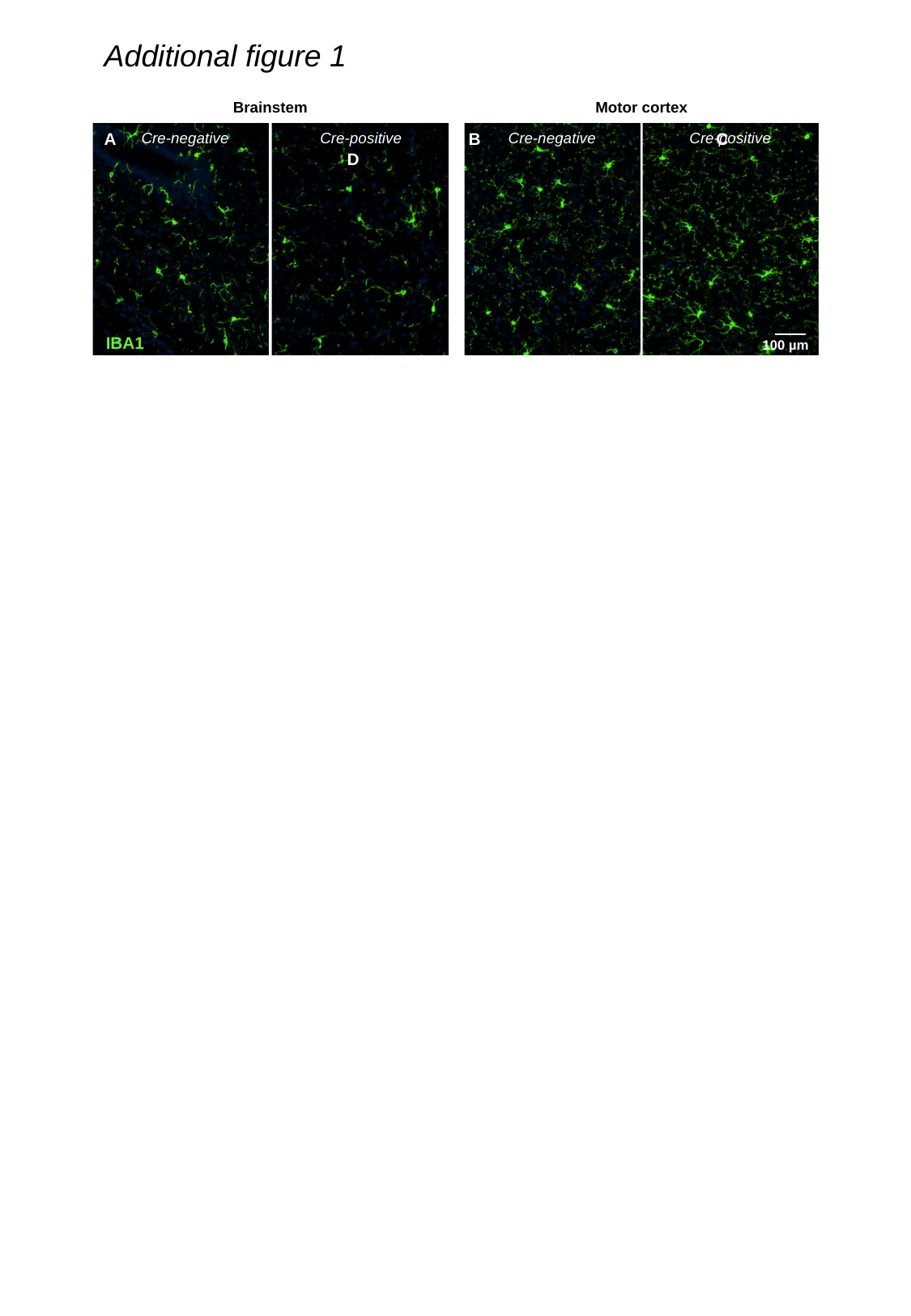

Additional figure 1
Brainstem
Motor cortex
A	 		B	 	 C	 		D
Cre-positive
Cre-negative
Cre-positive
Cre-negative
IBA1
100 µm
