# Supplementary figures and images for: Microglia lacking a peroxisomal β-oxidation enzyme chronically alter their inflammatory profile without evoking neuronal and behavioral deficits
Source: J Neuroinflammation. 2019 Mar 13;16:61. doi: 10.1186/s12974-019-1442-3 (PMC6417251; doi:10.1186/s12974-019-1442-3)

## Slide 1
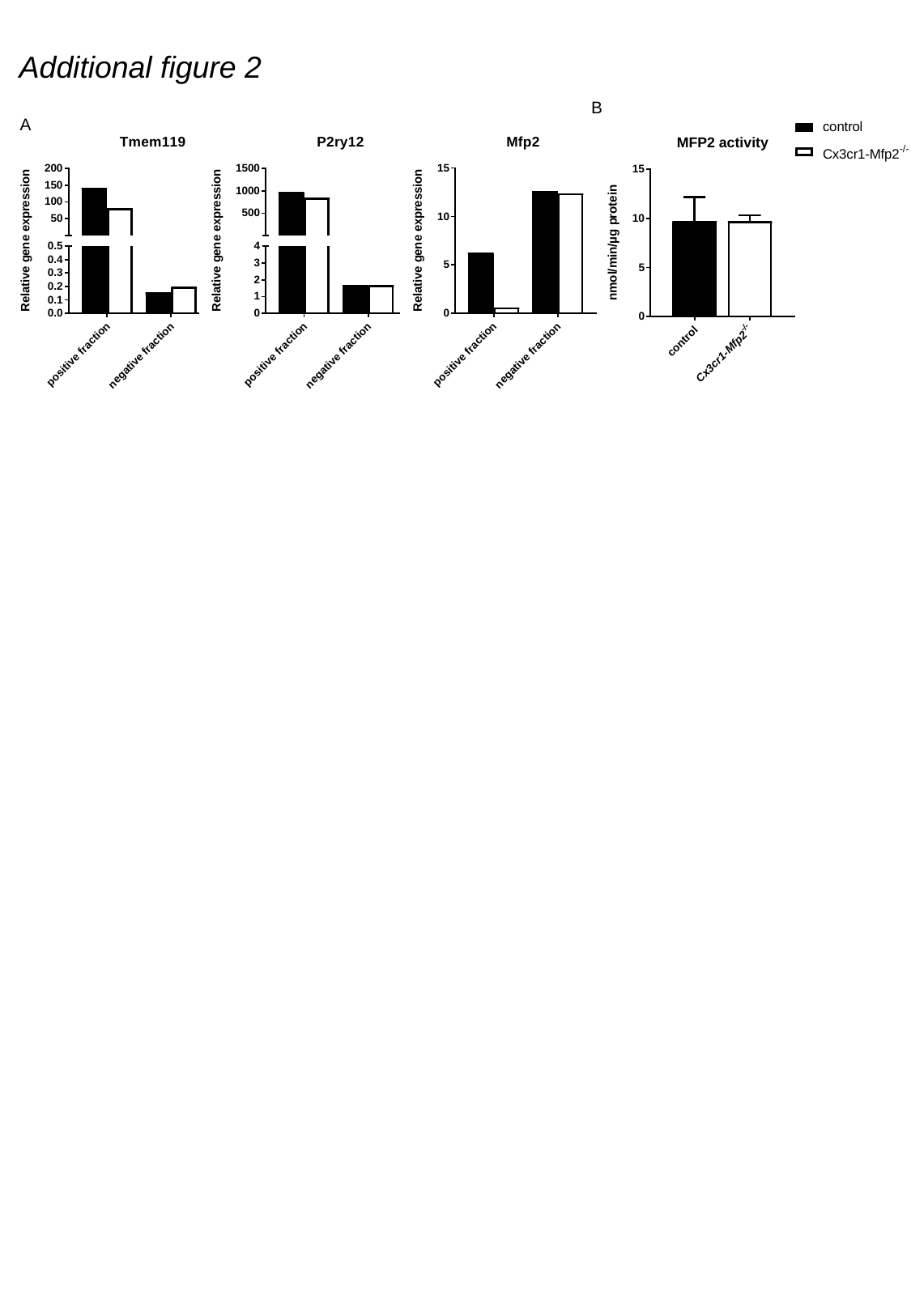

Additional figure 2
B
A

Supplement: Supplementary file 2 — Figure S2. Efficient and selective inactivation of MFP2 in microglia in Cx3cr1-Mfp2−/− mice. (A) Microglia were isolated from 11-month-old control and Cx3cr1-Mfp2−/− mice, and microglia purity was confirmed by the high expression of microglial markers (Tmem119 and P2ry12) in the positive (microglia) versus the negative fraction (neurons, astrocytes, and oligodendrocytes). Transcript expression of Mfp2 was determined in control and Cx3cr1-Mfp2−/− in both positive and negative fraction. Representative experiment out of two with similar results. (B) MFP2 activity in brain homogenates of control and Cx3cr1-Mfp2−/− mice. n = 2–3 mice/group. Mean ± SD is shown. (PPTX 46 kb) [file 12974_2019_1442_MOESM2_ESM.pptx]

## Slide 1
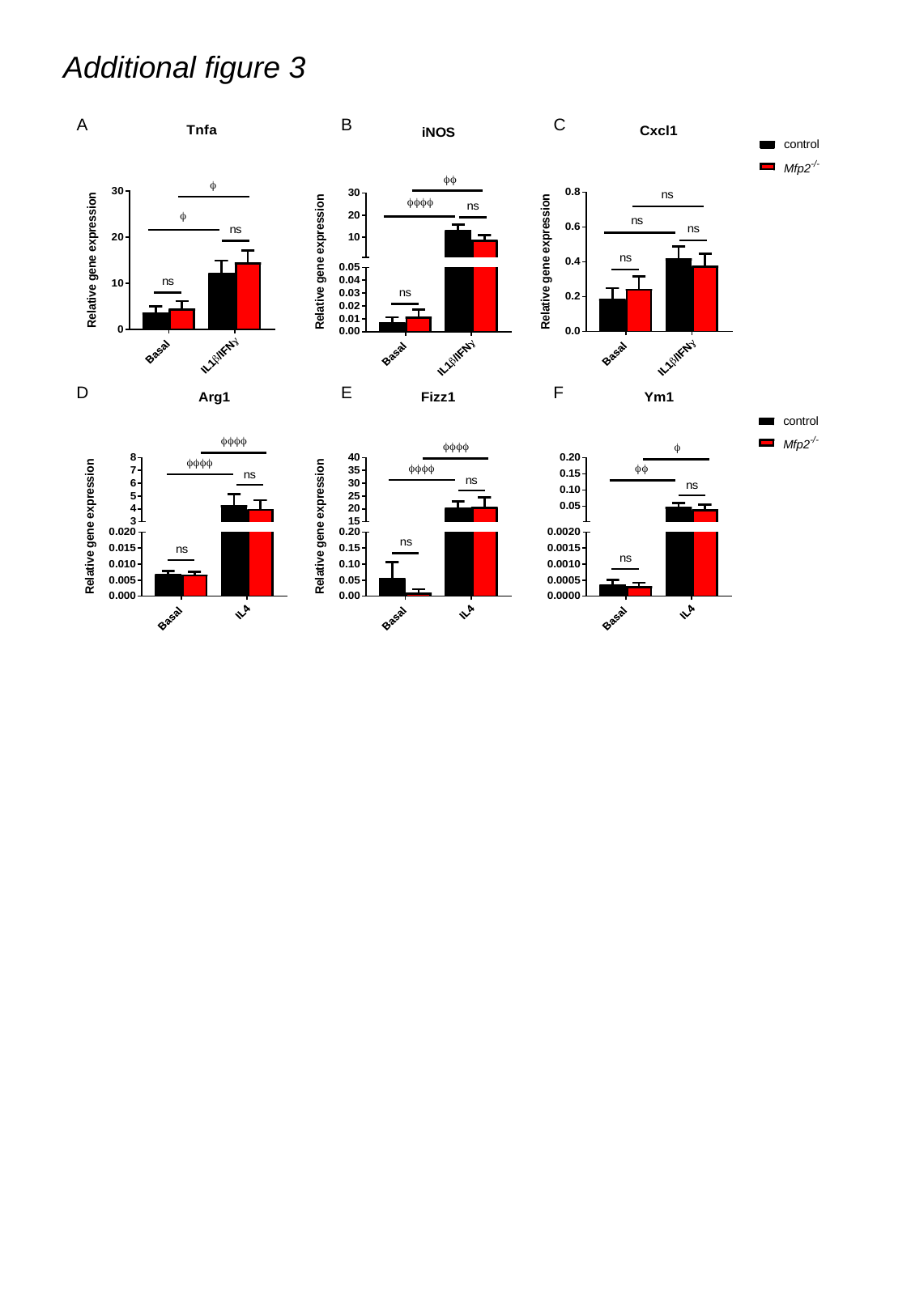

Additional figure 3
A
B
C
D
E
F

Supplement: Supplementary file 3 — Figure S3. Inflammatory properties of cultured Mfp2−/− and control microglia. MACS-isolated microglia from P8 mice were kept either in basal conditions or polarized to a pro-inflammatory state (Il1β/IFNγ) or an anti-inflammatory state (IL4). Transcript expression of pro-inflammatory (Tnfa, iNOS, Cxcl1) and anti-inflammatory cytokines (Arg1, Fizz1, Ym1) were determined. Significance levels: Φ p < 0.05, ΦΦ p < 0.01, ΦΦΦΦ p < 0.0001; ns, not significant. n = 8–11 mice/group. (PPTX 59 kb) [file 12974_2019_1442_MOESM3_ESM.pptx]
